# Cardiac ALL: Most Unusual Occurrence of Lenalidomide-associated Acute Lymphoblastic Leukemia with Subsequent Cardiac Involvement

**DOI:** 10.7759/cureus.6009

**Published:** 2019-10-28

**Authors:** Naman Sharma, Hani Hassoun, Joseph Hatem, Peter Kouides

**Affiliations:** 1 Internal Medicine, Rochester General Hospital, Rochester, USA; 2 Hematology / Oncology, Memorial Sloan Kettering Cancer Center, New York, USA; 3 Pathology, Rochester General Hospital, Rochester, USA; 4 Hematology / Oncology, Rochester General Hospital, Rochester, USA

**Keywords:** cardiac all, lenalidomide, secondary primary malignancy, inotuzumab, multiple myeloma

## Abstract

Leukemic infiltration of the myocardium is an extremely rare complication and requires high clinical suspicion, as <5% pf patients are symptomatic. Commonly encountered cardiovascular complications are secondary to anemia, infections, and chemotherapy.

We present an unusual case of biopsy-proven myocardial B-cell acute lymphoblastic leukemia (ALL) in an elderly male on chronic maintenance therapy with lenalidomide, with a previous history of multiple myeloma (MM) and subsequent ALL. Lenalidomide is a Category 1 recommendation for primary and maintenance therapy of MM, but there is growing evidence of secondary primary malignancies (SPMs). Despite this increase in SPM, the overall survival (OS) benefit associated with the use of maintenance immunomodulatory (IMID) therapy in multiple myeloma outweighs the risk of SPMs. Only age-appropriate screening methods are recommended.

This case report serves as an important reminder of a rare manifestation of leukemia and presents as anecdotal evidence of response to the monoclonal antibody inotuzumab for visceral involvement of ALL, which has not been reported to our knowledge and requires further exploration.

## Introduction

Leukemic infiltration of the myocardium is an extremely uncommon complication and only a few cases have been described in the literature [1-6]. Cardiovascular complications in leukemia are commonly due to anemia, infection, chemotherapy, and, rarely, secondary to leukemic infiltration of the myocardium, reported in 4% of secondary cardiac tumors [7]. Only <5% of cases are symptomatic and require high clinical suspicion.

We present an unusual case of biopsy-proven myocardial B-cell acute lymphoblastic leukemia (ALL) in an elderly male on chronic maintenance therapy with lenalidomide, with a previous history of multiple myeloma (MM) and subsequent ALL. Lenalidomide is a category 1 recommendation for primary and maintenance therapy of MM, but there is growing evidence of secondary primary malignancies (SPMs). Despite this increase in SPM, the overall survival (OS) benefit associated with the use of maintenance immunomodulatory (IMID) therapy in multiple myeloma outweighs the risk of SPMs. Only age-appropriate screening methods are recommended.

This case report serves as an important reminder regarding a rare manifestation of leukemia and presents as anecdotal evidence of response to the monoclonal antibody inotuzumab for visceral involvement of ALL, which has not been reported so far, to the best of our knowledge, and requires further exploration.

## Case presentation

An elderly male was diagnosed with multiple myeloma six years earlier and had received induction treatment with lenalidomide and dexamethasone followed by autologous stem cell transplantation and lenalidomide maintenance. While he achieved complete remission (CR) of his multiple myeloma, he developed progressive neutropenia, prompting a bone marrow biopsy that revealed B- cell ALL, with CD 19+, RUNX, and ETV-1 present. He underwent systemic therapy with hyper-CVAD, achieving complete remission (CR), followed by consolidation with allogeneic stem cell transplantation from his human leukocyte antigen (HLA) histocompatible brother. He remained in CR for a year followed by relapse, for which he received systemic therapy with blinatumomab, leading to complete remission with no evidence of minimal residual disease by flow cytometry.

**Figure 1:**
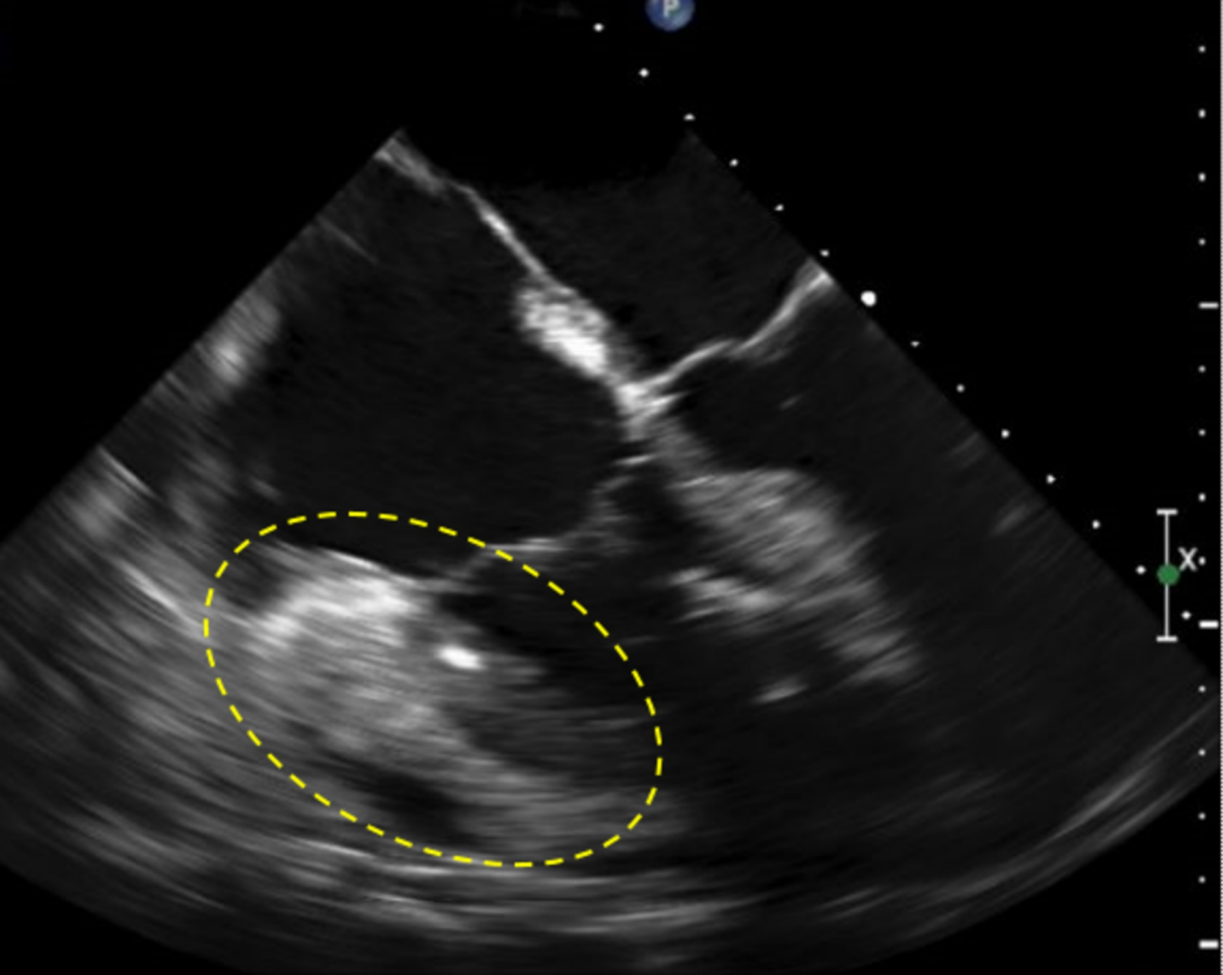
Transesophageal echocardiogram showing maximum RV thickening around the tricuspid annulus (dotted area) RV: right ventricle

After a disease-free interval of a year, the patient reported dyspnea on exertion. A surveillance bone marrow biopsy showed a recurrence of ALL with 10% blasts along with a minute abnormal plasma cell population detected by fluorescence-activated cell sorting (FACS). Fluorescent in-situ hybridization (FISH) showed two copies of ETV 6 (12p13) and four copies of RUNX 1 (21q22.3) detected in 1.3% of cells. He was shortly thereafter admitted in cardiogenic shock, and trans-esophageal echocardiogram (TEE) showed moderately reduced left ventricle (LV) systolic function with an ejection fraction (EF) of 35% and global hypokinesia (Figure 1). The right ventricle (RV) showed extensive echo density consistent with myocardial infiltration, more significant at the base around the tricuspid annulus along with pericardial effusion without tamponade physiology.

**Figure 2:**
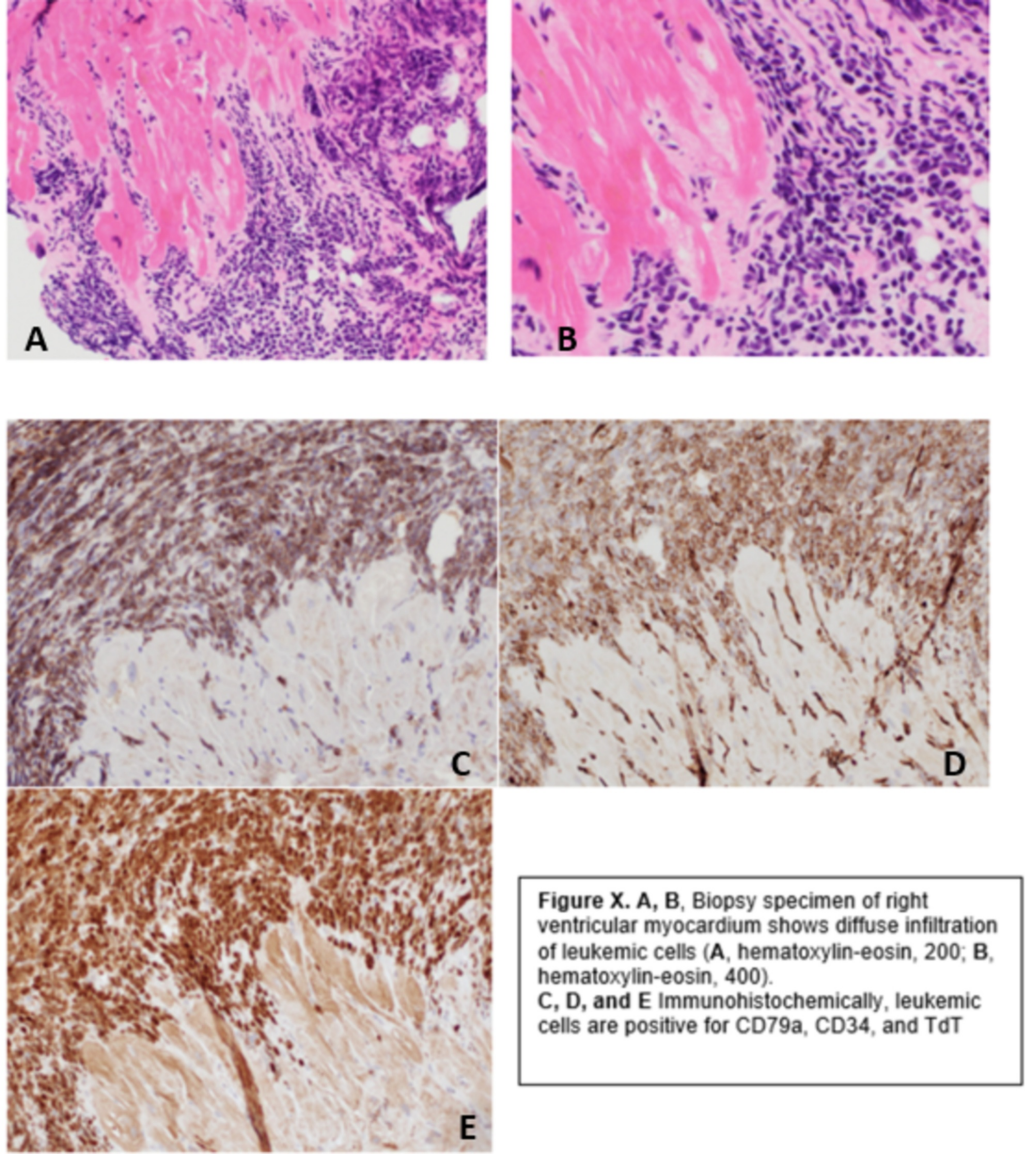
Immunohistochemical staining

A biopsy of the right ventricle mass showed fragments of the myocardium with infiltration by small to moderate-sized cells with fine chromatin, indistinct nucleoli, and scant cytoplasm. Single-cell apoptosis and scattered mitotic figures were seen. As seen in Figure 2, immunohistochemical stains demonstrated that the infiltrating cells were diffusely and strongly positive for the B-cell marker CD-79a and the precursor lymphocyte markers TdT and CD34, consistent with ALL infiltration.

The patient has shown considerable clinical improvement in cardiac function and is off continuous inotropic infusion after the second cycle of inotuzumab.

## Discussion

Leukemic cells can infiltrate any tissue but a clinically significant extra-medullary tumor in the heart has rarely been reported [1-6]. In general, the myocardium is an uncommon site of metastatic disease. In a 20-year autopsy study, cardiac metastases comprised only 1.2% of all cases of metastasis, amongst which leukemia constituted only 4% [7]. Cardiac involvement is commonly seen when the peripheral blast count is elevated and with advanced disease [8], but the presence of a high blast count is not always present when developing cardiac/tissue infiltration [9]. The clinical picture is variable, with the majority of patients being completely asymptomatic but others can present with chest pain, dyspnea, congestive heart failure, arrhythmia, ST-elevation, pericardial effusion, and tamponade [2,10]. Only <5% of patients are symptomatic, therefore, the diagnosis requires awareness and a low threshold of clinical suspicion.

An additional unusual and relevant aspect of this case is that our patient had multiple myeloma and was on prolonged maintenance therapy with lenalidomide for approximately six years and later was diagnosed with B-ALL, CD-19, RUNX, and ETV-1 positive.

Per the current National Comprehensive Cancer Network (NCCN) guidelines, lenalidomide is a Category 1 recommendation for primary and maintenance therapy, but there is a known risk of secondary primary malignancies (SPMs) with prolonged use. Several studies, including an analysis of pooled data from 11 clinical trials of lenalidomide-based therapy involving 3846 patients with relapsed refractory multiple myeloma (RRMM) reported an incidence ratio (IR) of 3.36 for all SPMs and 2.08 for all-invasive hematological malignancies [11]. Another retrospective study with 195 patients showed the incidence of myelodysplastic syndrome (MDS) and acute myelogenous leukemia (AML) as 1.29 and 1.08, respectively [12-13]. The overall median time of diagnosis of SPMs is 28 months for hematological and 15 months for solid tumors [13-14]. A case series by Tan et al. reported three cases of ALL in the setting of lenalidomide maintenance. In the reported cases, ALL developed after 2.5, three, and seven years of maintenance therapy [15]. Our case falls within this timeline. Certain factors associated with an increased risk of SPMs are the increased duration of therapy, prior treatment with alkylating agents, including autologous stem cell transplant and high-risk cytogenetics [16]. Despite this increase in SPM, the overall survival (OS) benefit associated with the use of maintenance immunomodulatory (IMID) therapy in multiple myeloma outweighs the risk of SPMs. However, there are no recommendations for additional tests other than the age-appropriate screening for malignancies in patients on lenalidomide maintenance therapy.

Our patient also had the chromosomal translocation t(12:21), resulting in the RUNX/ ETV fusion gene, which is the most frequent cytogenetic abnormality among patients with B-lineage childhood ALL, noted in 25% cases [17-18], and has been seen as early as in utero [19-20] and represents an initiating event in the development of childhood ALL. In itself, it is a weak cytogenetic abnormality and requires an additional genetic abnormality or “second hit” in order to develop full-blown leukemia [17] and the disease usually manifests in the initial years of life. In our case, it is unsure if the presence of this mutation predisposed the patient to the subsequent development of ALL with lenalidomide use. Another interesting observation is the response to the monoclonal antibody inotuzumab for visceral involvement of ALL, which has not been reported to our knowledge.

## Conclusions

In summary, our case serves as a reminder that ALL can develop on lenalidomide therapy for multiple myeloma and that relapsed ALL in the setting of being an SPM can involve the myocardium. Despite the advanced disease, there seems a potential use of inotuzumab for the treatment of visceral leukemia, which is yet to be explored.
